# Differential carbon source utilization drives metabolic state and resuscitation in antibiotic-tolerant persister cells

**DOI:** 10.3389/fphar.2025.1634627

**Published:** 2025-09-03

**Authors:** Yufei Sun, Sweta Roy, Qingbo Yang, Yinjie J. Tang, Dacheng Ren

**Affiliations:** ^1^ Department of Energy, Environmental, and Chemical Engineering, Washinton University in St. Louis, St. Louis, MO, United States; ^2^ Department of Biomedical and Chemical Engineering, Syracuse University, Syracuse, NY, United States; ^3^ Cooperative Research, College of Agriculture, Environmental and Human Sciences, Lincoln University of Missouri, Jefferson City, MO, United States; ^4^ Department of Civil and Environmental Engineering, Syracuse University, Syracuse, NY, United States; ^5^ Department of Biology, Syracuse University, Syracuse, NY, United States

**Keywords:** persister, isotopic tracing, resuscitation, *Escherichia coli*, CCCP, metabolism

## Abstract

**Introduction:**

Persistent infections remain challenging due to dormant bacterial cells that tolerate conventional antibiotics. Specifically, persister cells, phenotypic variants characterized by high antibiotic tolerance, can resume growth once antibiotic stress is alleviated. While general metabolic traits of persister cells have been documented, the metabolic shifts during persistence and resuscitation remain poorly understood.

**Methods:**

We applied stable isotope labeling using ^13^C-glucose and ^13^C-acetate to investigate metabolism in *Escherichia coli* persisters induced by carbonyl cyanide m-chlorophenyl hydrazone (CCCP). Labeling incorporation into metabolic intermediates and proteinogenic amino acids was measured using LC-MS and GC-MS.

**Results:**

The results demonstrated major differences in metabolic activities between normal and persister cells. Compared to normal cells, persister cells exhibited reduced metabolism. Peripheral pathways including parts of the central pathway, the pentose phosphate pathway, and the tricarboxylic acid (TCA) cycle, exhibited delayed labeling dynamics in persister cells. Proteinogenic amino acid profiling further demonstrated generalized but reduced labeling in persisters when using glucose as the sole carbon source, indicating a uniform slowdown in protein synthesis. Under acetate conditions, persister cells exhibited a more substantial metabolic shutdown, with markedly reduced labeling across nearly all pathway intermediates and amino acids. This reduction is likely due to substrate inhibition coupled with ATP demands required to activate acetate for central metabolism.

**Discussion:**

These findings help improve the understanding of bacterial persistence by demonstrating that persister cell metabolism adapts to available carbon sources. These insights into persister metabolism may inform the development of targeted strategies to more effectively combat persistent bacterial infections.

## 1 Introduction

Persistent bacterial infections remain challenging due to the resilience of persister cells, which are capable of surviving antibiotic treatments by entering a dormant state (Balaban et al., 2019). Conventional antibiotics were selected for inhibition of bacterial growth; thus, persister cells exhibit a high level of tolerance to a wide range of antibiotics. These persister cells pose significant hurdles in treatment regimens, often leading to recurrent infections after antibiotic treatment (Van den Bergh et al., 2017). The inefficiency in antibiotics toward persistent infections highlights the urgent need for novel therapeutic approaches specifically designed to target and eliminate persister cells.

It is well accepted that persister cells are growth arrested; however, there have been debates about the metabolic state of persister cells and whether they are dormant or metabolically active (Wood et al., 2013; Prax et al., 2014; Kim et al., 2016; Kim et al., 2017; Rahman et al., 2024). Recent research has begun to shed light on the metabolic adjustments that persister cells undergo during antibiotic exposure. Investigations into the metabolic state of persister cells have revealed that these cells have diminished cellular functions, such as lower energy levels, suppressed transcriptional and translational activities, all of which contribute to the emergence of persister cells (Mohiuddin et al., 2024; Niu et al., 2024). These cellular attributes significantly enhance tolerance to antibiotics, allowing persister phenotype within bacterial communities (Lewis, 2007; Wood et al., 2013; Pu et al., 2019; Wilmaerts et al., 2019). Single-cell analyses have revealed that persister cells exhibit markedly reduced growth rates or enter a non-replicative state (Zhang et al., 2024), indicative of a dormancy phase (Balaban et al., 2004). For example, in *Staphylococcus aureus*, a diminished cellular energy level has been associated with the downregulation of critical enzymatic activities in tricarboxylic acid (TCA) cycle (Wang et al., 2018; Zalis et al., 2019). Moreover, transcriptomic analyses of isolated persisters have identified a widespread downregulation of genes pivotal for energy production and essential cellular functions, highlighting a global reduction in metabolic activity (Shah et al., 2006; Keren et al., 2011; Peyrusson et al., 2020). Furthermore, interventions such as pre-treatment of bacterial populations with transcription inhibiting antibiotics like rifampicin, have been shown to significantly increase the proportion of persister cells (Kwan et al., 2013; Kim et al., 2016; Wood, 2016). It was observed that exposure to rifampicin converted all *Escherichia coli* cells into persisters (Kwan et al., 2013; Kim et al., 2016; Wood, 2016) indicating a physiological shift towards dormancy. Previous proteomic studies have provided insights into the physiological state of persister cells, demonstrating significant alterations in protein expression associated with persistence, including the downregulation of proteins involved in energy metabolism, protein synthesis, and cellular growth processes, along with the activation of proteins responsible for stress responses and DNA damage repair (Hu et al., 2015; Li et al., 2015; Truong et al., 2016; Windels et al., 2019). Such proteomic adjustments were observed both in the presence and absence of antibiotics, reinforcing the notion that persister cells inherently possess metabolic adaptations essential for maintaining dormancy and enhancing survival under stressful conditions. While studies have demonstrated the dormant nature of persisters, there have also been reports indicating metabolic activities in persister cells (Prax et al., 2014; Rahman et al., 2024). The discrepancies are partially caused by the differences in experimental setup, both in the procedure to generate and isolate persister cells, and the assays conducted. Many studies to date are based on characterization of mutant stains, transcriptomics, and proteomics. While these are powerful tools, they do not directly measure the function of metabolic pathways. Deeper insights are needed to analyze the enzyme activities in central pathways that are essential for the survival of persisters and maintenance of the persister state.

In this study, we employed ^13^C-glucose and ^13^C-acetate to trace the metabolic pathways in *E*. *coli* persister cells induced by carbonyl cyanide m-chlorophenyl hydrazone (CCCP) compared to uninduced controls. Unlike indirect approaches like transcriptomics or proteomics, ^13^C labeling of fast turnover metabolites via LC-MS can rapidly delineate functional pathways (Li et al., 2017; Czajka et al., 2020), revealing the metabolic state of persister cells. However, the natural abundance of persister cells is extremely low, presenting a significant challenge to obtain sufficient biomass (Dewachter et al., 2019). To overcome this limitation, the induction of persister populations through chemical pretreatment has been widely adopted (Sulaiman et al., 2018; Huemer et al., 2021). Among various inducing agents, we selected CCCP, a protonophore that disrupts proton gradients and ATP synthesis without permanent damage to essential cellular processes, providing a consistent and reversible induction of persisters suitable for metabolic studies (Cavari et al., 1967; Kwan et al., 2013). The results from our study highlighted the altered metabolic fluxes and identified key changes in persister cells induced by membrane depotentiation. The results demonstrated significant shifts in the utilization of these substrates, emphasizing the flexibility and adaptability of bacteria in response to stresses that lead to dormancy. These insights can help develop targeted therapies that disrupt these adaptive mechanisms, potentially leading to more effective eradication of persistent bacterial infections.

## 2 Materials and methods

### 2.1 Bacterial strain and culture medium

*Escherichia coli* BW25113 (Baba et al., 2006) was routinely cultured in Lysogeny broth (LB) (Sambrook et al., 1989) containing 10 g/L NaCl, 5 g/L yeast extract, and 10 g/L tryptone or M9 medium containing 100 mL of 5X M9 salts, 1 mL of 1 M MgSO_4_, 50 μL of 1 M CaCl_2_ in 500 mL H_2_O supplemented with 2 g/L glucose.

### 2.2 CCCP induced persister cells

An overnight culture of *E. coli* BW25113 in M9 medium containing 2 g/L glucose was sub-cultured in the same medium with a starting OD_600_ of 0.05 at 37 °C with shaking at 200 rpm. When the subculture reached OD_600_ of 0.5, cells were immediately exposed to 100 μg/mL of CCCP for 15 min at 37 °C with shaking at 200 rpm to induce persister formation. After persister induction, cells were collected by centrifugation at 13,000 rpm for 3 min at room temperature and then washed three times in M9 medium without any carbon source.

### 2.3 Tracer experiments with ^13^C-glucose or ^13^C-acetate

Control and induced CCCP persister cells were washed and concentrated to OD_600_ of 5 in 10 mL of M9 medium. To avoid the effects of dead cells/cell debris on metabolic flux measurements, we chose CCCP over antibiotics to induce persister formation prior to ^13^C labeling. The cells were immediately labelled with 2 g/L 1,2–^13^C_2_ glucose (CLM-504-PK) or 2 g/L 2–^13^C sodium acetate (CLM-381-PK) from Cambridge Isotope Laboratories, Inc. In M9 medium and incubated at 37 °C with shaking at 200 rpm at specific timepoints (0, 20 s, 5 min, 30 min and 2 h). At each specific timepoint, samples were quickly cooled by liquid nitrogen to stop metabolic activities within a few seconds, then the resulting sample (temperature ∼0 °C) was centrifuged at 4 °C and 5,000 × g for 3 min. The quenched cell pellets were stored at −80 °C. Each experimental condition was tested with three biological replicates. For ^13^C metabolite analysis, the cell pellet was first lyophilized. Then 0.5 mL extraction solution of 80:20 methanol-water was added to the lyophilized biomass and incubated at −20 °C for 1 h, followed by centrifugation at 10,000 × g for 10 min at 0 °C. The resulting supernatant was then filtered with a 0.2 µm filter and transferred to autosampler vials for LC/MS analysis. The remaining cell pellets were then treated with 1.5 mL 6 N HCl at 100 °C for 18 h to hydrolyze the proteins. The hydrolyzed proteinogenic amino acids were analyzed by the TBDMS method (Hollinshead et al., 2016). For ^13^C Metabolite tracing with LC/MS, the extracted free metabolites were analyzed by a ThermoFisher Q-Exactive LC-MS system. The method was modified from a previous protocol (Czajka et al., 2020). To a m/z scan range of 40–900. The sample was analyzed using an Agilent Technologies InfinityLab Poroshell 120 HILIC-Z column (2.1 × 100 mm, 2.7 µm) with an Agilent InfinityLab Poroshell 120 HILIC-Z guard column (2.1 × 5 mm, 2.7 µm). GC-MS and LC-MS data were analyzed using Agilent ChemStation Enhanced Data Analysis software and Maven, respectively.

### 2.4 Persister count and growth curve of resuscitated persister cells

To determine the number of persister cells induced by CCCP, cells were collected after CCCP induction by centrifugation at 13,000 rpm for 3 min at room temperature and washed three times with phosphate buffered saline solution (PBS). Ten μg/mL of ofloxacin was added to the washed cells and incubated for 5 h at 37 °C with shaking at 200 rpm to kill any remaining normal cells. After incubation, cells were collected by centrifugation and washed with PBS three times. The cells were then resuspended in PBS and plated on LB agar plates to count CFU using the drop plate method (Sieuwerts et al., 2008). Each experimental condition was tested with three biological replicates. For analysis of growth of resuscitated persister cells, persister cells were washed and resuspended in 200 μL M9 medium supplemented with either 2 g/L glucose or 2 g/L acetate to an initial OD_600_ of 0.2. The samples were then transferred to a 96 well plate and incubated at 37 °C with shaking at 200 rpm. OD_600_ was recorded every 30 min for 40 h using an Epoch 2 Microplate Spectrophotometer (BioTek, Winooski, VT, USA).

## 3 Results

### 3.1 Persister cell formation and resuscitation

**FIGURE 1:**
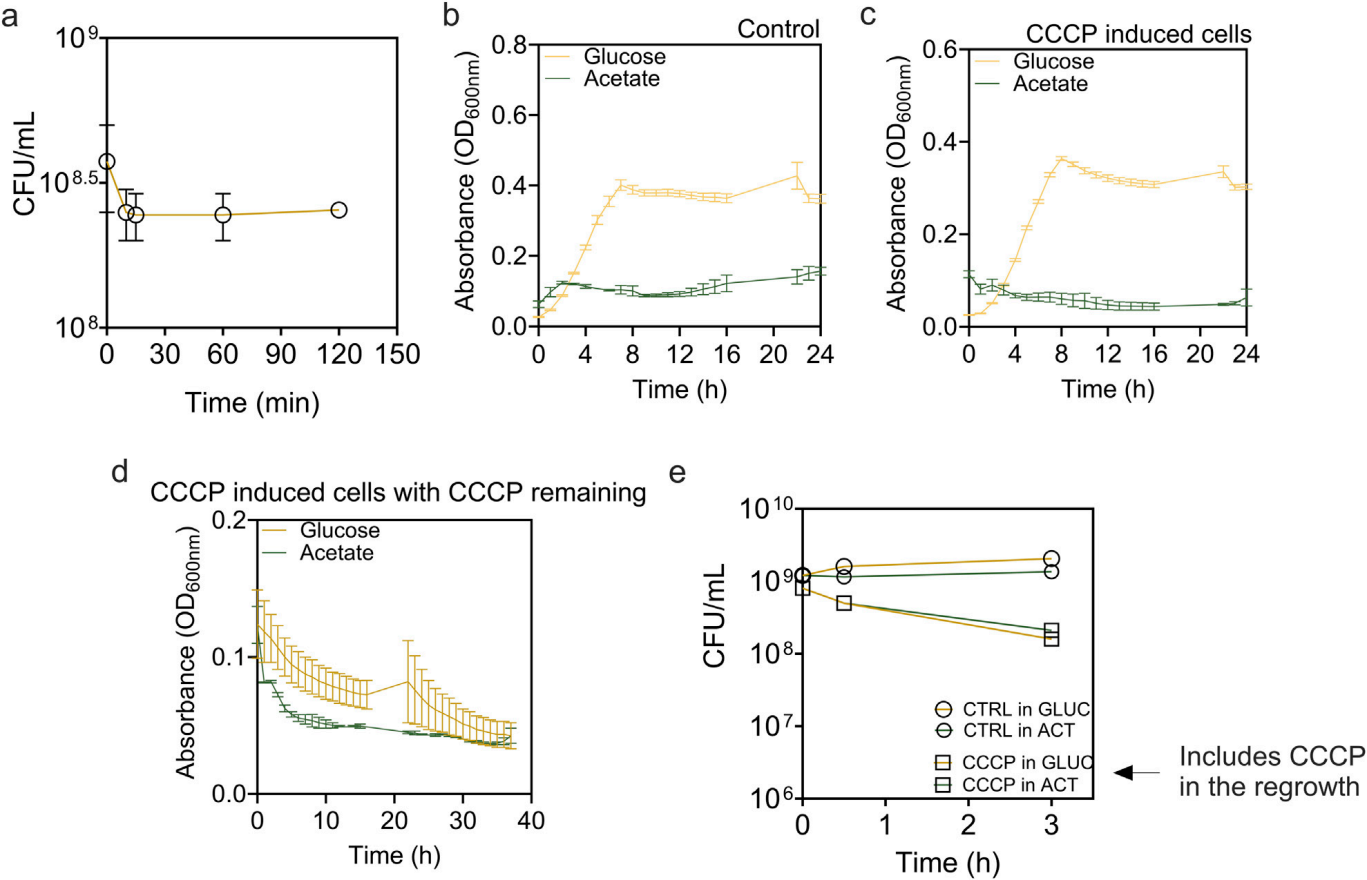
Formation and resuscitation of *E. coli* BW25113 persisters induced by CCCP. **(a)** Time dependent killing curve of *Escherichia coli* BW25113 exposed to 100 μg/mL of CCCP for 0–120 min. After treatment, cells were washed and viability was determined based on CFU. **(b–d)** Growth curves of **(b)** exponential phase cells, **(c)** CCCP induced cells, and **(d)** CCCP-induced cells in the continuous presence of CCCP. Cells were incubated in M9 medium supplemented with 2 g/L glucose or 2 g/L acetate as indicated. **(e)** Viability of control cells and CCCP-induced cells in the continuous presence of CCCP during incubation.

**FIGURE 2:**
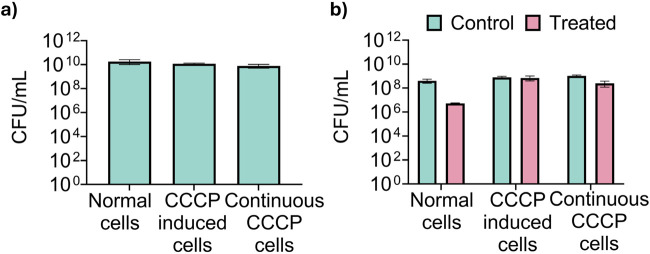
Validation of CCCP-induced persister cells for metabolic tracing. **(a)** Total CFU of untreated exponential phase *Escherichia coli* BW25113 cells (labeled as “normal cells”), cells treated with 100 μg/mL CCCP for 15 min (“CCCP induced persister cells”), and cells treated with CCCP and maintained in 100 μg/mL CCCP during regrowth (“Continuous CCCP cells”) prior to metabolic labeling. **(b)** Viability of normal cells, CCCP induced persister cells, and CCCP induced cells in the continuous presence of CCCP after treatment with 10 μg/mL of ofloxacin for 5 h in PBS to determine persister count.

To induce persister formation, we treated *E. coli* BW25113 cells with CCCP in M9-glucose medium. The amount of persister cells was quantified after exposing cultures to CCCP for 15–120 min (Figure 1a). The short-time exposure to a high concentration of CCCP (100 μg/mL for 15 min) resulted in a highly enriched persister population (∼91%), as demonstrated by high survival rate after subsequent ofloxacin treatment (Figure 2b). Our use of a higher CCCP concentration for a shorter period in minimal M9 media induced rapid metabolic arrest without extensive cell death, providing sufficient biomass for ^13^C labeling. The minimal medium also reduced ^13^C tracing noises from unknown nutrients in rich media. The results show that the proportion of persisters remained consistent across the extended exposure time. We also observed the regrowth of the induced cells in M9 medium supplemented with 2 g/L glucose or 2 g/L acetate (Figures 1b,c).

To understand what metabolic activities are present (if any) in persister cells, we added either ^13^C-labeled glucose or ^13^C-labeled acetate to persister cells and conducted detailed metabolic flux analysis. Using ^13^C-labeled glucose allows us to trace primary metabolic pathways essential for energy production and recovery. Conversely, ^13^C-labeled acetate was tested to explore persister cell metabolism under suboptimal conditions because acetate requires more complex metabolic adaptations and does not support rapid regrowth, reflecting the capability of persister cells to resuscitate in less favorable environments. The results demonstrated that while both exponential *E. coli* cells (labeled as normal cells) and CCCP-induced persister cells were able to resume growth in the presence of glucose, they failed to do so with acetate as the sole carbon source, highlighting the critical role of carbon source in recovery (Figures 1b,c). Furthermore, our results also indicated that within 2 h of shifting to fresh medium, CCCP induced persister cells were still in lag phase in the presence of glucose. To ensure that persister cells retained their persister state during metabolic flux analysis, we monitored the regrowth of CCCP-induced cells in the continuous presence of CCCP (Figure 1d). Our findings indicated no significant growth but continued decrease in OD_600_ instead, suggesting that cells were unable to restore metabolic activity under continuous stress from CCCP. This result is corroborated by the viability assay (Figure 1e), which showed that in the continuous presence of CCCP, cell viability declined markedly, with 40%–50% of cells losing viability even in the presence of carbon source of glucose or acetate. Thus, the presence of CCCP provided a good condition for evaluating metabolic state of persister cells without resuscitation.

After validating the persister state, we proceeded with metabolic labeling of cells using ^13^C-glucose and ^13^C-acetate to trace metabolic activities in these cells. Our experimental setup included three conditions: exponential phase cells, CCCP-induced cells with CCCP washed out, and CCCP-induced cells with CCCP present during the labeling process. Time course samples were collected at intervals of 20 s, 1 min, 5 min, 30 min, and 2 h post-labeling to monitor metabolic integration and flux changes. The number of viable cells was consistent among the samples as shown in Figure 2a. In addition, the number of persister cells was quantified for each sample by exposing cells to 10 μg/mL ofloxacin for 5 h as we described previously (Dhaouadi et al., 2024). The results indicated that CCCP induced high-level persister formation (Figure 2b).

### 3.2 Metabolic dynamics of glucose utilization in persister cells

**FIGURE 3:**
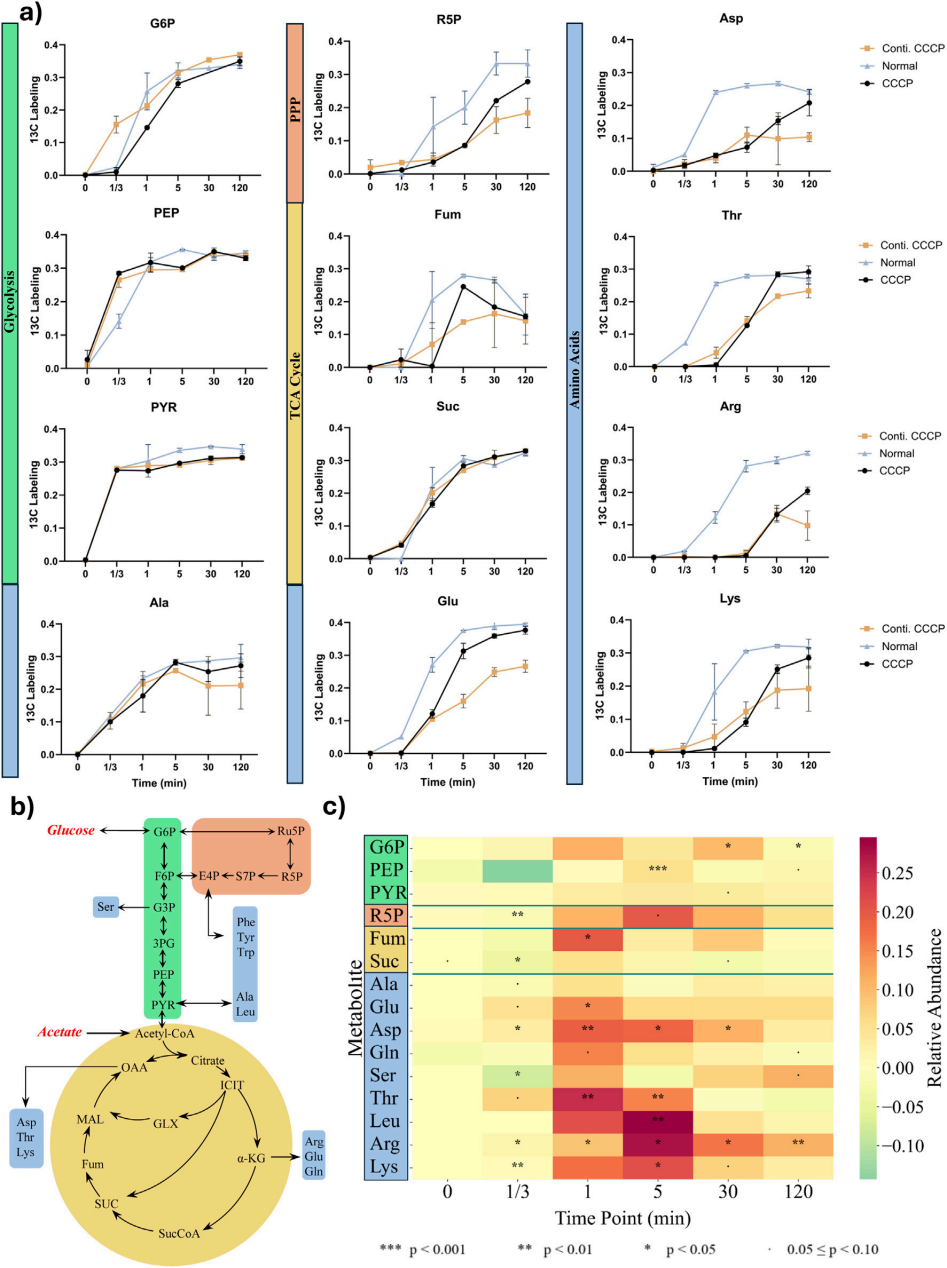
Metabolite labeling fraction through each pathway using glucose as substrate. **(a)** ^13^C fraction of each detected metabolite over time. Metabolites are categorized into Glycolysis (Green), Pentose Phosphate Pathway (Orange), TCA Cycle (Yellow), and Amino acids (Blue). **(b)** Pathway Scheme of central metabolic pathway including amino acids with utilization of glucose and acetate. **(c)** Heatmap of ^13^C-labeling differences between normal and persister cells. Values represent labeling in normal cells minus persister cells (*ΔLabeling = Normal − Persister*). Positive values indicate higher enrichment in normal cells. Intracellular amino acids ranked in the order of approximate biosynthetic depth. The results were analyzed with two -sample *t*-test to determine statistical significance (n = 3).

The incorporation rates of ^13^C-labeled glucose into key metabolites revealed distinct differences between normal and CCCP-induced persister cells. Initial labeling rates for early glycolytic intermediates, glucose-6-phosphate (G6P), and phosphophenyl pyruvate (PEP), were comparable across both cell populations (Figure 3a), indicating that glycolysis remains active in CCCP-induced persisters. The limited early labeling observed in these metabolites suggests rapid turnover and low intracellular pool sizes, rather than impaired pathway activity.

In contrast, significant delays in labeling were observed for intermediates of the pentose phosphate pathway (PPP), ribose-5-phosphate (R5P) and the tricarboxylic acid (TCA) cycle metabolite fumarate, particularly evident from 1 min to 120 min post-labeling (Figure 3a). These delays suggest metabolic bottlenecks within these catabolic pathways during persistence. Amino acid synthesis displayed varied kinetics. Alanine, directly derived from pyruvate, showed labeling kinetics similar in persisters and actively growing cells, indicating that core metabolic functions are retained in these cells. However, amino acids with more biosynthesis enzyme steps, such as lysine, arginine, threonine, leucine, and aspartate, demonstrated pronounced delays in label incorporation (Figures 3b,c), indicating substantial impairment in the biosynthesis of complex amino acids during the persister state. Despite these initial delays, relative abundances of key metabolites gradually aligned between persisters and normal cells at later time points, suggesting that persisters maintain baseline metabolic activity for essential biosynthetic processes. Collectively, these findings support the concept that bacterial persister formation involves targeted metabolic downregulation rather than a complete cessation of all enzyme activities (Conlon et al., 2016; Rahman et al., 2024; Bollen et al., 2025). However, as the stress continues, it is possible that the active metabolites (such as cofactors and ATP) and functional enzymes will be gradually depleted/deactivated, randering the cells into a deeper dormancy state and form viable but nonculrable cells (VBNCs) and eventially cell death. VBNC cells fail to grow in standard culture media, making them unculturable under common conditions. In contrast, persister cells are a dormant subpopulation that can survive antibiotic exposure and retain the ability to regrow once stress is removed. While both cell types exhibit high tolerance to antibiotics and environmental stress, a key distinction lies in culturability and resuscitation dynamics (Ayrapetyan et al., 2015). Testing this hypothesis is part of our ongoing work.

### 3.3 Acetate metabolism

**FIGURE 4:**
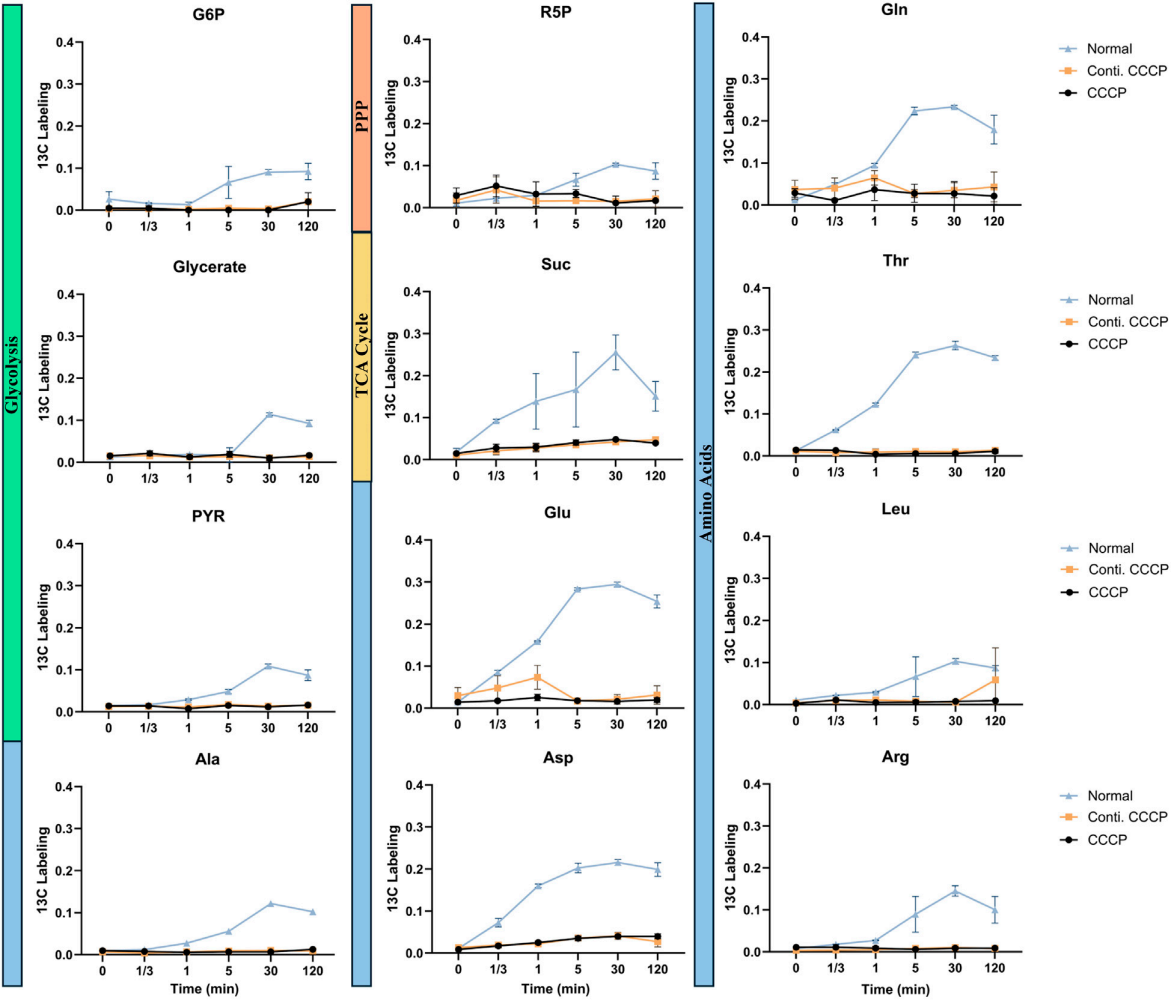
Metabolite labeling fraction through each pathway using acetate as substrate. ^13^C fraction of each detected metabolite over time. Metabolites are categorized into Glycolysis (Green), Pentose Phosphate Pathway (Orange), TCA Cycle (Yellow), and Amino acids (Blue). Data represent mean ± standard deviation of biological triplicates (n = 3).

When acetate was used as the carbon source, normal cells displayed increased delays in metabolite labeling compared to the glucose results above. Acetate is a less energy efficient substrate compared to glucose and metabolic regulatory networks are affected by the uptake rate of carbon sources from the environment (Basan et al., 2020; Hartline et al., 2022; Zhang et al., 2024). Consistently, a significant metabolic shutdown was observed in CCCP-induced persister cells across nearly all analyzed pathways (Figure 4). The incorporation of ^13^C from acetate into central metabolites, as well as amino acids, was largely reduced, indicating inhibition in metabolic activity under acetate-based resuscitation conditions.

The observed differences in metabolic activity between acetate and glucose utilization are likely due to their distinct uptake and metabolism mechanisms. Unlike glucose, which enters the cell via phosphotransferase system (PTS) (Abernathy et al., 2019), acetate uptake primarily relies on passive diffusion and acetyl-CoA synthetase (ATP + Acetate + CoA → AMP + Pyrophosphate + Acetyl-CoA). This entry route consumes significantly more ATP compared to glucose glycolysis with net ATP generations. Moreover, the assimilation of acetyl-CoA typically proceeds through the glyoxylate shunt and TCA cycle, requiring additional enzymatic steps for biosynthesis pathways such as gluconeogenesis. Acetate metabolism is further constrained by regulatory controls, substrate inhibition, and thermodynamic reaction bottlenecks, collectively rendering acetate catabolism inherently slower and less energetically efficient compared to glucose (Xiao et al., 2013). Meanwhile, persister cells typically exhibit physiological adaptations such as altered membrane composition (Rowlett et al., 2017), increased cell wall rigidity (Uzoechi et al., 2020), and reduced membrane permeability (Allison et al., 2011). Such changes could notably limit the diffusion of acetate into cells. While acetate uptake occurs via passive diffusion, the modified cell envelope structures of persister cells could impose additional diffusion barriers, significantly slowing acetate penetration and utilization (Xiao et al., 2013). Consequently, this limited substrate availability at the intracellular level could amplify the observed metabolic impairment and severely restrict acetate incorporation into central metabolic pathways and biomass synthesis in these dormant cells.

### 3.4 Proteinogenic amino acid analysis

**FIGURE 5:**
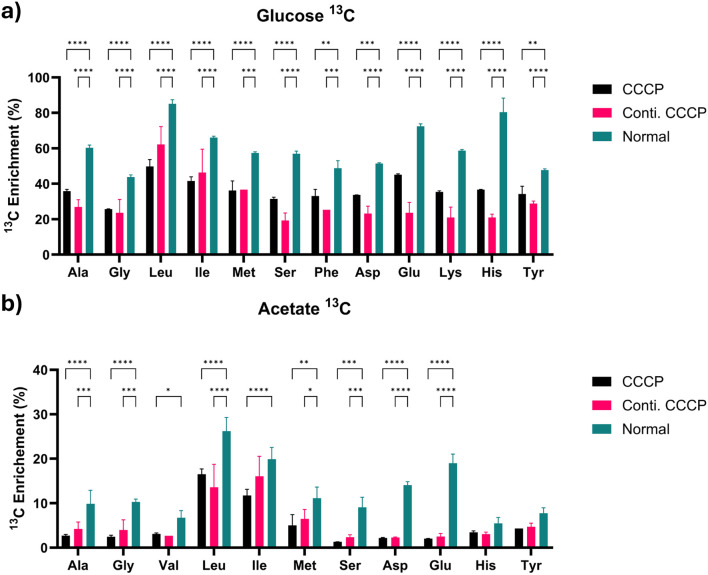
Proteinogenic amino acids analysis at 2 h with **(a)** glucose or **(b)** acetate as substrate. Values indicate relative enrichment of ^13^C substrate (adjusted value based on the initial isotopic substrates fraction, 0.33 and 0.50, respectively, for 1,2–^13^C_2_ glucose and 2–^13^C acetate). Data are shown as mean ± SD (n = 3). Statistical analysis was performed using two-way ANOVA followed by Tukey’s *post hoc* test. *P* < 0.05 (*), p < 0.01 (**), p < 0.001 (***), *p* < 0.0001 (****). Asterisks indicate statistically significant differences between treatment groups.

The labeling in protein amino acids indicates active anabolism. Proteinogenic amino acid analysis revealed notable differences in ^13^C labeling patterns between glucose and acetate conditions (Figures 5A,B). In glucose conditions, hydrolyzed proteins from CCCP-induced persister cells displayed a significant, albeit consistently lower, incorporation of ^13^C labeling compared to normal cells. This uniformly observed effect across all amino acids suggests a generalized slowdown in overall protein synthesis during persistence, possibly reflecting a conserved strategy to reduce energy consumption and resource allocation under stress conditions.

The addition of acetate created a significant drop in labeling fractions, with most metabolites reaching less than 10% enrichments within persister cells, demonstrating substantial metabolic impairment. This reduction results from the limited metabolic entry points and additional metabolic demands for acetate assimilations, coupled with reduced enzyme activity and expression levels in persister cells. Interestingly, leucine and isoleucine maintained relatively higher labeling levels compared to other amino acids despite showing a slight reduction from normal cells. This may be attributed to their biosynthesis pathways having a closer metabolic link to TCA cycle intermediates, allowing relatively easier access to acetate-derived carbon under stress conditions.

These results demonstrate that persister cells exhibit substrate-dependent metabolic changes overall with slower biosynthetic pathway progression when they undergo resuscitation. The observed metabolic diversity demonstrates how bacterial cells adjust their metabolic flow to maintain survival and recovery capabilities when facing different environmental stress situations. Better understanding of these adaptive changes may help identify metabolic targets that can be leveraged to create more effective persister control methods.

## 4 Discussion

This study provides an in-depth, quantitative view of metabolism during initial resuscitation of *E. coli* persister cells. By coupling ^13^C tracing with two chemically and bioenergetically distinct substrates, glucose and acetate, we were able to track carbon flow through central and peripheral pathways with minute-scale resolution and reveal substrate-contingent bottlenecks that classic single-substrate MFA could not capture. Persister cells formed during normal cultures are low abundant, hindering MFA analyses that require large amounts of biomass (Dewachter et al., 2019). To overcome this challenge, we employed short-time exposure to CCCP, which effectively induces persister formation, allowing sufficient biomass for detailed metabolic studies (Sulaiman et al., 2018; Huemer et al., 2021). Although this method does not entirely replicate natural conditions under which persisters arise, it provides a robust and practical approach to studying persister cell metabolism. The majority of the cells (∼91%) in our treated samples were indeed persisters, based on antibiotic susceptibility. It is worth noting that the other 9% of the CCCP-treated population were no longer regular normal cells. As the observed labeling differences substantially exceed the ratio of CCCP-induced vs. normal cells (Figures 3, 4). There are multiple conditions that CCCP-treated cells do not have any detectable activities (Figure 4). Thus, the other 9% of the cells were also entering dormancy as persister-like cells. This indicates that the method used in this study can effectively detect metabolic profiles of persister cells. Further studies at single cell level will help reveal more specific changes and the heterogeneity in related populations. This is part of our ongoing work.

It is important to note that metabolic activities depend on the presence of functional enzymes, not necessarily cell growth or viability, as demonstrated in cell-free synthesis (Garenne et al., 2021). Although persister cells are dormant and lack major metabolic activities, our data confirms that CCCP induced persister cells do have the essential enzymes needed to resuscitate back that can deliver ATP, NAD(P)H, and precursor metabolites to avert lethal damage and resuscitate upon favorable changes in their environment. Rapid, near-maximal labeling of early glycolytic intermediates (G6P, PEP) within 60 s demonstrates that upper glycolysis remains competent and may serve as an ATP “jump-starter” for glucose utilizations once conditions improve. Downstream throttling of the PPP and TCA cycle limits metabolic activities such as respiration and endogenous reactive oxygen species (ROS) production—a process associated with antibiotic lethality (Brynildsen et al., 2013). Therefore, controlled restriction of these pathways may serve as a redox-protective adaptation.

Our data also uncovered a clear hierarchical pattern in amino acid biosynthesis recovery, closely tied to metabolic complexity and energetic demands. Amino acids synthesized through direct and energetically inexpensive reactions, such as alanine (derived from pyruvate), aspartate (from oxaloacetic acid), and glutamate (from α-ketoglutarate), rapidly achieved more than 80% enrichment within the first minute of resuscitation. This indicates prioritization of low-cost biosynthetic pathways crucial for immediate cellular repair and macromolecular maintenance. In contrast, amino acids that require long, energy-demanding routes, including the branched-chain group (leucine, isoleucine, valine), the basic amino acids arginine and lysine, and the aromatics, gained label much more slowly. In persisters, labeling of these compounds remained markedly lower than in exponential-phase controls in the 5- and 30-min samples, and approached similar levels only by the 120-min point.

This timing pattern is consistent with a two-step recovery process (Wilmaerts et al., 2019). During the initial phase of resuscitation, cells appear to prioritize low-cost building blocks that support immediate macromolecular maintenance and cofactor replenishment. Once internal energy and redox status are presumably restored, and the stringent alarmone ppGpp is likely reduced, the cells can reactivate longer, ATP-demanding pathways required for full biosynthesis and renewed growth. This could help avoid premature energy drain and limit the extra reactive-oxygen stress that a sudden surge in TCA-cycle activity could impose, thereby smoothing the transition out of dormancy. Although we did not measure ATP, NAD(P)H, or ppGpp directly, the labelling kinetics we observed fit well with this stepped recovery model, which can be examined more fully in future studies that combine flux tracing with direct measurements of these intracellular signals.

Compared to glucose, acetate metabolism exhibited a marked difference between normal and persister cells: incorporation of carbon from acetate into the TCA cycle and downstream metabolites remained below five percent even after 2 hours, contrasting sharply with the rapid labeling observed in exponentially growing cells during the same period. This limitation begins at initial activation step, where acetyl-CoA synthetase converts acetate into acetyl-CoA, consuming two ATP equivalents. The minimal isotopic enrichment observed indicates that persister cells lack sufficient ATP reserves to sustain this energetically demanding reaction, which is consistent with current literature (Wilmaerts et al., 2018; Spanka et al., 2019; Huemer et al., 2021; Pandey et al., 2021). In addition, there could be a lack of active enzymes associated with dormancy stage that can also impact the minimal isotopic enrichment observed in persister cells. Many studies have indicated that during dormancy, enzymes involved in translation and metabolism can become sequestered into protein aggregates (Pu et al., 2019; Huemer et al., 2021), further contributing to reduced metabolic activity in persister cells. Furthermore, acetate substrate inhibition may exacerbate this energetic constraint. Acetate primarily enters the cell through passive diffusion; however, persister cells typically exhibit reduced membrane permeability due to increased rigidity (Uzoechi et al., 2020). Such membrane adaptations likely slow the entry of acetate and other polar nutrient molecules, thereby further contributing to broader delays observed in the labeling of several amino acid pools. Collectively, the high ATP requirement for acetate activation and the possible impaired transport across structurally altered membranes establish dual metabolic and physical barriers to persister cell recovery. Future experiments combining ^13^C isotope tracing with direct quantification of intracellular ATP, acetyl-CoA levels, and membrane fluidity assessments will help test this hypothesis.

This study still has limitations. Due to technical constraints associated with rapid capturing metabolic profiles at the scale of seconds to minutes, our tracer experiments did not physically separate the remaining cells in a population with different depths of dormancy, potentially introducing measurement noise. For instance, glycolytic enzymes, even in cell-free systems, can support a thermodynamically favorable flux from glucose to pyruvate (Abernathy et al., 2019). However, the magnitude of metabolic differences observed significantly exceeds the estimated proportion of cells that were becoming dormant but were not antibiotic tolerant persisters yet (9%), and the acetate results showed even more profound differences. These results indicate that our method is effective in monitoring persister-related metabolic changes. In future work, integrating metabolic analysis with transcriptomics, single-cell redox sensors, and cell sorting will refine our understanding of how energy flow, signaling networks, and cell subpopulations during persistence. It is also important to note that the persisters in this study were induced by a short-time exposure to CCCP. Longer exposure to stresses or unfavorable substrates could render the cells enter a deeper dormancy state and most enzymes may be depleted. Understanding the effects of such changes on metabolisms is part of our future work.

In summary, our dual-substrate isotopic tracing qualitatively depicts a modular metabolic architecture in *E. coli* persisters. The overall metabolism is reduced in persister cells, consistent with their dormant nature. Although core ATP-generating glycolysis remains agile at the persister state, auxiliary biosynthetic and energy-intensive pathways are throttled according to substrate quality and pathway depth. These findings help understand bacterial dormancy and provide insights for metabolism-informed therapeutic strategies against persistent infections. From a translational perspective, metabolic potentiators that boost intracellular ATP (thereby forcing premature exit from dormancy) could be paired with existing antibiotics for “wake-and-kill” regimens. In addition, isotope-guided screening may identify metabolites or pathways that selectively disrupt persister-specific flux nodes without perturbing commensal metabolism.

## Data Availability

The raw data supporting the conclusions of this article will be made available by the authors, without undue reservation.
